# Early lactation performance in primiparous and multiparous women in relation to different maternity home practices. A randomised trial in St. Petersburg

**DOI:** 10.1186/1746-4358-2-9

**Published:** 2007-05-08

**Authors:** Ksenia Bystrova, Ann-Marie Widström, Ann-Sofi Matthiesen, Anna-Berit Ransjö-Arvidson, Barbara Welles-Nyström, Igor Vorontsov, Kerstin Uvnäs-Moberg

**Affiliations:** 1 Department of Woman and Child Health, Division of Reproductive and Perinatal Health Care, Karolinska Institutet, Stockholm, Sweden; 2 Department of Hospital Pediatrics, St. Petersburg State Pediatric Medical Academy, St. Petersburg, Russia; 3 Department of Animal Environment and Health, Swedish University of Agricultural Sciences, Skara, Sweden

## Abstract

**Background:**

There are not many studies exploring parity differences in early lactation performance and the results obtained are fairly often contradictory. The present study investigated the effect of different maternity home practices in St. Petersburg, Russia, as well as of physiological breast engorgement and maternal mood, on milk production in primi- and multiparous women on day four. The amount of milk was studied in relation to the duration of "nearly exclusive" breastfeeding.

**Methods:**

176 mother-infant pairs were randomised into four groups according to an experimental *two-factor design *taking into account infant *location *and *apparel*. Data were recorded in the delivery ward at 25–120 minutes postpartum and later in the maternity ward. Group I infants (n = 37) were placed skin-to-skin in the delivery ward while Group II infants (n = 40) were dressed and placed in their mother's arms. Both groups later roomed-in in the maternity ward. These infants had the possibility of early suckling during two hours postpartum. Group III infants (n = 38) were kept in a cot in the delivery and maternity ward nurseries with no rooming-in. Group IV infants (n = 38) were kept in a cot in a delivery ward nursery and later roomed-in in the maternity ward. Equal numbers per group were either swaddled or clothed. Episodes of early suckling were noted. Number of breastfeeds, amount of milk ingested (recorded on day 4 postpartum) and duration of "nearly exclusive" breastfeeding were recorded. Intensity of breast engorgement was recorded and a Visual Analogue Scale measured daily maternal feelings of being "low/blue".

**Results:**

On day four, multiparas had lower milk production than primiparas when they were separated from their infants and breastfeeding according to the prescriptive schedule (7 times a day; Group III). In contrast, there was no difference in milk production between multi- and primiparous mothers in the groups rooming-in and feeding on demand (Groups I, II and IV), although multiparas had higher numbers of feedings than primiparas. In addition during the first three days postpartum, multiparous mothers had higher perception of physiological breast engorgement and lower intensity of feeling "low/blue" than primiparous mothers. Early suckling was shown to positively affect milk production irrespective of parity. Thus Group I and II infants who suckled within the first two hours after birth ingested significantly more milk on day 4 than those who had not (284 and 184 ml respectively, SE = 14 and 27 ml, p = 0.0006).

Regression analyses evaluated factors most important for milk production and found in Groups I and II for primiparous women that early suckling, intensity of breast engorgement and number of breastfeeds on day 3 were most important. Intensity of feeling "low/blue" was negatively related to amount of milk ingested. The significant factor for multiparous women was early suckling. Similar results were obtained in Groups III and IV; however, in primiparous mothers, engorgement was the most important factor and in multiparous women it was rooming-in. Amount of milk produced on day 4 was strongly correlated to a duration of "nearly exclusive" breastfeeding (p < 0.0001).

**Conclusion:**

The present data show that ward routines influence milk production. As our data suggest that milk production in primi- and multiparous women may be differently influenced or regulated by complex factors, further research is needed.

## Background

Maternity practices focusing on care routines of mother and infant after birth have changed greatly over the last decades due, in part, to recommendations deriving from research. One such example of this change is the practice of putting the newborn baby directly in skin-to-skin contact with the mother immediately after birth. The reasons for skin-to-skin contact are many: it may enhance early breastfeeding, as the baby has been shown to have an inborn pre-feeding behavioural instinct and starts suckling the breast by him/herself [1]; the mother keeps the baby warm [2] and the baby will be relaxed [3] and in a non-crying state [4]. Studies have also shown that rooming-in [5,6] and breastfeeding on demand [7] are favourable for the establishment of lactation. This knowledge has encouraged WHO and UNICEF to include these findings in their joint statement to maternity units worldwide to protect, promote and support breastfeeding [8].

The present study, conducted from 1995 to 1998, is part of a collaborative Russian – Swedish longitudinal research project which evaluated the effect of maternal-infant care routines in Russian maternity homes. More than 99% of women give birth in freestanding maternity homes in Russia. At the time of this study, typical care routines included infants' being swaddled immediately after birth according to cultural traditions [9], separation of mothers and babies, infants cared for in a nursery, breastfeeding at seven regularly scheduled times per day. Furthermore, partners were not allowed in the hospital during labour and birth, nor during the postpartum period.

At the onset of this project no official statistics about the duration of exclusive breastfeeding were available in Russia; however, in St. Petersburg, it was estimated that approximately 95% of mothers initiated breastfeeding while residing in the maternity homes. The duration of any breastfeeding was estimated to be 64% at 3 months, 31% at 6 months and 10% at 9 months (personal communication, Igor Vorontsov, Chief paediatrician of St. Petersburg). Exclusive breastfeeding was unusual.

The research team became interested in evaluating the effects of some of these care practices on mothers and infants. Primarily we studied effects of delivery ward practices on the baby's body temperature [3]. Secondly, we focused on the effects of Russian ward practices on breastfeeding parameters and infant weight loss during the first days of life [10]. In the present study we focus on differences in early lactation between primiparous and multiparous women. We have observed earlier (unpublished observations) a slight difference in breastfeeding competence in multiparas compared to primiparas and we thus decided to focus this investigation on whether or not there were any differences in breastfeeding characteristics between mothers as related to parity, maternal physiological breast engorgement and maternal postpartum mood.

Previous studies have demonstrated that the amount of breast milk increases significantly with the degree of physiological breast engorgement [11], and the pattern and extent of physiological engorgement has been reported to be a possible predictor for sufficient milk supply [12].

Changes in mood ("maternity blues") are well-known phenomena coinciding in time with the initiation of lactation. One of the main current hypotheses explains the blues as a result of activation of brain structures connected with parenting, where oxytocin is believed to play a central role [13]. As far as oxytocin is involved both in breastfeeding outcome and in changes in mood after birth it was hypothesized that milk production could be indirectly affected by ward practices through its probable influence on mother's mood.

In a randomised trial using an experimental factorial design, we studied the effects of skin-to-skin contact versus contact in clothes and separation at the delivery ward, rooming-in versus nursery care and swaddling versus baby clothes at the maternity ward as well as the mother's mood and her perception of physiological breast engorgement on the amount of breast milk produced/ingested at day four after birth. These factors were studied in relation to the duration of "nearly exclusive" breastfeeding in primi- and multiparous women.

## Methods

This study took place from 1995–1998 in St Petersburg, Russia at Maternity Home 13, which is a freestanding maternity home. It has approximately 2100–2300 births per year; the standard length of stay for new mothers is five postpartum days. Ethnographic data were collected about care routines for mothers and infants. Description of material and methods is also given elsewhere [3,10].

### Inclusion criteria

Recruitment of participants took place on regular weekdays during daytime hours. When a healthy woman, free of chronic disease and with an uncomplicated pregnancy was admitted to the delivery ward, she was informed about the study. If she intended to breastfeed and had no preference about rooming-in, swaddling, and skin-to-skin contact, she was asked if she was willing to participate. Women who gave informed consent and had a normal non-instrumental delivery without oxytocin infusion and/or analgesia such as epidural, paracervical, or pudendal block, and a healthy baby (not small for gestational age, without congenital malformations and with Apgar score not less than 8 at five minutes after birth) were included in the study.

**Figure 1:**
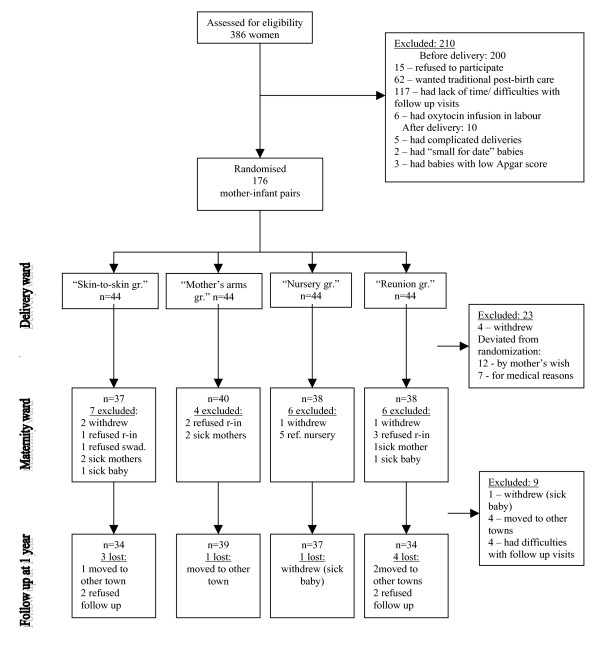
**Flow diagram**. Definition of the treatment groups: *"Skin-to-skin group" *– comprised of infants lying skin-to-skin on their mother's chest 25–120 minutes after birth at the delivery ward with later rooming-in at the maternity ward. *"Mother's arms group" *– comprised of dressed infants lying on their mother's chest 25–120 minutes after birth at the delivery ward with later rooming-in at the maternity ward. *"Nursery group" *– comprised of dressed babies kept in a cot in a labour ward nursery 25–120 minutes after birth and remained at the maternity ward nursery without rooming-in with their mothers. *"Reunion group" *– comprised of dressed babies kept in a cot in a labour ward nursery 25–120 minutes after birth, but then taken to the maternity ward for rooming-in with their mothers.

During the four years of data collection, a total of 386 women were assessed for eligibility to participate in the study. Two hundred and ten mothers declined or were not included for different reasons (Figure 1).

### Randomisation and subjects

A table of allocation sequence was drawn up in advance of the trial. For the purpose of balancing the probable influence of time and parity, the future mother-infant pairs were grouped in sets of eight consecutive dyads, separately for primiparas and multiparas. The randomisation to the eight treatment combinations was performed in blocks of eight mothers independently of the other blocks. In total there were 11 blocks of 8 primiparas and 11 blocks of 8 multiparas. Opaque envelopes containing the information on group assignment were sealed and numbered in the order they should be used: primi- 1, 2, 3 . . . 88, and multi- 1, 2, 3 . . . 88. Both the researchers and the recruited women were blind to the task.

A total of 176 mother-infant pairs were randomly assigned according to allocation sequence. Immediately after the infant was born and the condition of the mother and baby was assessed as normal, the envelope (with the lowest number) coinciding with parity with information on group assignment was opened. During the approximate first 25 minutes after birth, all infants were subjected to identical compulsory Russian labour ward routines (see description below). After that the infants were treated according to the randomisation grouping.

The experimental design used in this study represents a *two-factor design *(Table 1). The first factor was *"Baby's location" *which took into account where the infant was located in respect to one of four location combinations (*Location I–IV*) as specified below by group. The second factor was "*Apparel*" and took into account if the baby was swaddled (*Apparel 1*) or dressed in clothes (*Apparel 2*). The two factors combined gave a total of 4 × 2 treatment combinations or four main groups.

**Table 1:** Experimental design

***Main groups***	*Delivery ward*		*Maternity ward*	
	
	**Location**	**Apparel**	**Location**	**Apparel**
I "Skin-to-skin group" n = 37	Skin-to-skin	No	Rooming-in	Swaddling
	Skin-to-skin	No	Rooming-in	Clothes
II "Mother's arms group" n = 40	Mother's arms	Swaddling	Rooming-in	Swaddling
	Mother's arms	Clothes	Rooming-in	Clothes
III "Nursery group" n = 38	Nursery	Swaddling	Nursery	Swaddling
	Nursery	Clothes	Nursery	Clothes
IV "Reunion group" n = 38	Nursery	Swaddling	Rooming-in	Swaddling
	Nursery	Clothes	Rooming-in	Clothes

Group I *(Location I)*: The "Skin-to-skin group" was comprised of infants (n = 37) lying skin-to-skin on their mother's chest 25–120 minutes after birth, while still in the delivery ward. After this period, they were swaddled (*Apparel 1*) or put in clothes (*Apparel 2*) and taken to the maternity ward for rooming-in with their mothers and to breastfeed on demand. Group II *(Location II)*: The "Mother's arms group" was comprised of babies (n = 40) who were swaddled (*Apparel 1*) or put in clothes (*Apparel 2*) and lying on the mother's chest 25–120 minutes after birth, who were then taken to the maternity ward for rooming-in with their mothers and to breastfeed on demand. Group III *(Location III)*: The "Nursery group" was comprised of babies (n = 38) who were swaddled *(Apparel 1) *or put in clothes *(Apparel 2)*, but kept in a cot in the labour ward nursery 25–120 minutes after birth. These infants did not room-in with their mothers but remained in the nursery at the maternity ward, except for when they were taken into the mother's room for breastfeeding seven times per day. Group IV *(Location IV)*: The "Reunion group" was comprised of infants (n = 38) who were swaddled *(Apparel 1) *or in clothes *(Apparel 2) *and kept in a cot in the labour ward nursery 25–120 minutes after birth, but who were then taken to the maternity ward for rooming-in with their mothers and to breastfeed on demand.

### Excluded mothers

Of the 176 women who were assigned to the study, 23 were excluded for a variety of reasons during the stay at the maternity ward (Figure 1). Four mothers withdrew from the study, without giving a reason; twelve did not want to follow the randomisation (five women switched from the nursery to rooming-in; six women switched from rooming-in to nursery; and one woman wanted another type of attire for the baby than that to which he was assigned); seven mother-infant pairs were excluded because of medical complications. These were: maternal bleeding (four exclusions), maternal heart problem (one exclusion), and health problems with the infant, hyperbilirubinemia caused by blood incompatibility (two exclusions).

### Background variables

The final number of mother-infant dyads within the randomised groups divided by parity is shown in Table 2 and Table 3. Other variables shown on these tables include maternal age, duration of labour, the infants' gestational age and birth weight, presented as means and standard deviations, and in addition the number of episiotomies and infants' gender.

**Table 2:** Clinical characteristics of the primiparous mothers and their newborn babies in four groups; means and (SD)

	Mother's age (years)	Mother's education (years)	Duration of labour (hours)	Episiotomy (n)	Gestational age (weeks)	Babies' birth weight (g)	Gender, girls (n)
Group I (n = 21)	23.9 (4.8)	14.3 (2.3)	9.2 (3.0)	12	39.3 (0.8)	3342 (423)	10
Group II (n = 20)	23.0 (3.5)	13.3 (2.2)	9.4 (3.9)	12	39.5 (1.1)	3407 (485)	8
Group III (n = 20)	25.5 (3.5)	14.0 (2.6)	8.9 (3.1)	17	39.6 (0.8)	3346 (368)	10
Group IV (n = 20)	23.3 (3.0)	13.7 (2.2)	9.8 (3.7)	14	40.0 (1.0)	3448 (456)	8

**Table 3:** Clinical characteristics of the multiparous mothers and their newborn babies in four groups; means and (SD)

	Mother's age (years)	Mother's education (years)	Duration of labour (hours)	Episiotomy (n)	Gestational age (weeks)	Babies' birth weight (g)	Gender, girls (n)
Group I (n = 16)	29.8 (5.0)	14.0 (2.9)	8,1 (2,6)	8	39.4 (1.2)	3743 (376)	6
Group II (n = 20)	31.9 (5.0)	13.8 (2.5)	7.2 (2.4)	8	39.7 (0.9)	3479 (487)	14
Group III (n = 18)	31.3 (5.1)	13.8 (3.2)	6.2 (2.6)	8	39.4 (0.9)	3532 (440)	7
Group IV (n = 18)	30.7 (4.3)	14.2 (2.2)	6.2 (1.9)	6	39.8 (1.0)	3689 (469)	7

All mothers were either married or lived with a partner (four couples cohabited). The educational background did not differ between primi- or multiparous women, or between the groups. All multiparous women had previous breastfeeding experience.

There were no significant differences between the 153 women who participated in the study and the 23 women who were excluded in respect to their educational background or other background variables as described above (Table 2 and Table 3). Furthermore, since cigarette smoking had been considered as a potentially interesting variable in respect to breastfeeding outcome, it was also assessed. However, since only 6 mothers smoked 3–10 cigarettes per day and the smokers were evenly distributed between the groups, smoking was not taken into account in the analysis.

### Routines at the delivery and maternity wards

Some of the more important routines included mothers receiving intravenous administration of methylergometrin when the infant's head had emerged from the birth canal. The infant's umbilical cord was clamped 10–15 seconds postpartum and immediately after that all infants were placed onto an examination table where they were carefully dried, wrapped in a dry cotton sheet and left under a radiant heater while the attending midwife took care of the new mother. Infants were then washed under tap water by a midwife, who then weighed the baby, took anthropometric measurements, and applied a drop of 30% sulfacyl natrium to the conjunctiva for prophylaxis of gonococcal ophtalmia. Xeroform powder or 1% iodine solution was applied to the umbilical cord stump and on the infants' skin folds to prevent infection.

After these obligatory Russian routines, the infants were, according to the randomisation, given to their mothers or placed in a cot in the nursery (dressed or swaddled). Babies given to their mothers were placed either naked skin-to-skin on the mother's chest between her breasts, or placed directly in the mothers' arms (dressed or swaddled).

If there was no bleeding in the women, the suturing of spontaneous vaginal ruptures and episiotomies was postponed until two hours after delivery. At two hours postpartum, a paediatrician examined infants and then, according to the randomisation schema, the babies were taken to the maternity ward for rooming-in with the mothers or were taken to the nursery and placed in a cot.

### Swaddling

**Figure 2:**
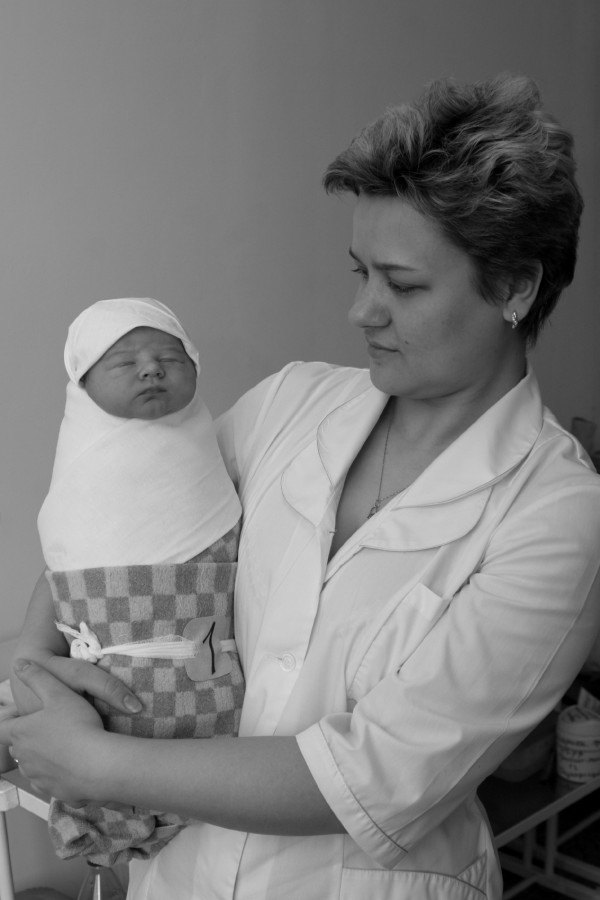
A photo of Russian swaddled baby.

Swaddling is a traditional Russian practice of infant care, and is used on babies shortly after birth until the infant and mother are discharged from the maternity home. Swaddling then is continued for some months. In the maternity home the following procedure was used: two cotton cloths were placed between the baby's legs, as a diaper, and another cloth was wrapped around both of the infant's legs. The next cloth was used to wrap the baby's arms close to the body, serving as an extra layer of covering of the body and legs. After this, one more cloth was wrapped from behind the head to cover the forehead, which ended cross-wise on the thorax. The final cloth, placed inside the outer blanket, was then wrapped tightly around the entire body of the infant (see Figure 2).

### Baby's clothes

**Figure 3:**
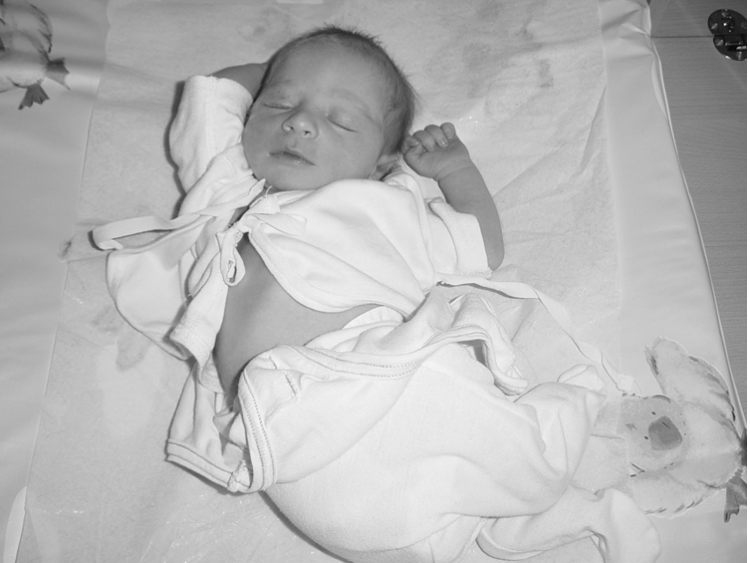
A photo of Swedish baby in clothes.

Babies who were randomised to wear clothes were dressed in identical sets of infant wear, specially brought from Sweden for the purpose of the study. Each set consisted of a loose cotton shirt with long sleeves, leggings, woolen socks and a cap and infants were dressed in this apparel after the birth (see Figure 3). Disposable nappies were used.

### Variables measured

Early suckling. In the groups where the babies stayed with their mothers for two hours at the delivery ward, mothers could initiate breastfeeding. These early breastfeeding episodes were observed and it was noted if the baby attached to the breast and suckled with distinct sucking movements.

At the maternity ward the mothers filled in a diary for each day (if the baby was born after noon, this day was defined as "day 0"). The mothers assessed a variety of factors: breast engorgement, the mother's perception of tension/hardness in the breasts [14] as "none", "slight" or "marked"; feeling "low/blue", by a mark on a 100 mm long horizontal Visual Analogue Scale (VAS) with the end points " not at all" and "as much as I can imagine" (the English expression "feeling low/blue" was translated into Russian and retranslated by an other person into English without language confusion); and number of breastfeeds per 24 hours. 

Milk intake. On day 4 (i.e. in the interval between 72 and 96 hours after delivery), the amount of breast milk ingested by the baby was measured as the difference in weight before and after each feeding (Electronic scale, Tanita 1581, Tanita Corporation, Japan). If the breastfeeding session began before 72 hours postpartum and was finished after 72 hours, the breastfeed was not taken into consideration. In case a suckling episode started before 96 hours postpartum, but finished after this time, it was included in the calculation. The mothers performed the weighing of infants if they were rooming-in, while nurses weighed the babies kept in the nursery. The scales in the nursery and in the mother's room were of the same model, specially purchased for the study. Each mother was educated by the staff about how to weigh the baby and checking for accuracy during weighing episodes. Milk ingested, originally considered as a difference in weight pre- and post-breastfeeding, was also considered and reported as milk production, as well as milk volume, in some analyses. The volume of supplemental formula and/or glucose was measured and noted.  

"Nearly exclusive" breastfeeding was assessed over a year-long period at specific follow-up times at the maternity home (at 1, 2, 3, 4, 6, 9 and 12 months after birth). Prior to the measurement days, a research coordinator for the project phoned each mother to fix the closest to exact date day and time of the visit. During the follow-up visits a paediatrician examined the babies and anthropometrical measurements were taken. Mothers were asked about breastfeeding and whether or not they used any supplementation. Exclusive breastfeeding, i.e. breastfeeding without any supplementation, is not a common practice in Russia, and thus could not be registered in this study. Instead the expression used in this study was *"nearly exclusive breastfeeding" *which was defined as breastfeeding including *irregular *supplementation of juices or solids in a daily volume of 30 ml or less, or formula in a daily volume of 100 ml or less [15].

All mother-infant pairs except nine were followed up until the age of 12 months. Among those, four had unexpectedly moved to other towns and four mothers had difficulties visiting the maternity home for follow-up. One infant became ill at 1 month of age (intracerebral haemorrhage) and the mother withdrew (see Figure 1)

### Analysis

Means and standard errors (SE) were used as descriptive measures if not otherwise specified. To compare the treatment groups, one-way ANOVA or Kruskal-Wallis tests were used. Kruskal-Wallis test was used instead of ANOVA when comparing groups with few observations and risk of non-normality in distributions. Repeated measures ANOVA together with Fisher's Protected Least Significant Differences (Fisher's PLSD) were used to analyse data over time [16].

Multiple linear regression was used to explore the relationship between the variable "milk amount on day 4" in a series of exploratory regression analyses, using different combinations of the explanatory variables, including early suckling, breast engorgement, feeling "low/blue", number of breastfeeds at day 3, and the design variables skin-to-skin or not, rooming-in or not, and swaddling or not. For use in the regression analyses, the variable "engorgement" – originally classified into three groups, "none", "slight" and "marked" – was re-coded into a 0 – 1 dummy variable by coding "none" and "slight" to 0 and "marked" to 1. The variable "feeling low/blue" was re-coded for the regression analysis as "low" when under 40 and "marked" when 40 or more was noted on a Visual Analogue Scale (VAS). This break point (40 on a VAS) had been chosen to discriminate mothers with marked "feeling low/blue" according to the distribution (approximately 15% of all mothers in the study).

Many of the analyses in this study are performed on data from day 3, the day before the amount of breast milk was measured. Additionally, since the randomisation was stratified by parity, separate analyses for primiparas and multiparas were possible.

Sample size was originally determined by using experiences from previous studies basing power calculations on variation obtained in these studies. To check the adequacy of the sample size retrospectively we used the variance observed in the present study and found that to compare groups the most probability to detect difference of 75 ml of ingested/produced milk is about 66% and to detect difference of 100 ml is about 97% which for instance is the difference on milk amount if early suckling or not occurred.

Data were treated with "per protocol" analyses and not according to "intention to treat" as the present study has an explanatory, rather than a pragmatic aim [17].

### Ethical considerations

Prior to conducting the study, approval was granted by the Ethics Committee of the Karolinska Institutet, Stockholm, Sweden, and the Health Care Division of the Mayor-Council of St. Petersburg, Russia.

## Results

### Effect of parity and ward routines

#### Milk volume – day 4

When the average milk volume ingested by infants was measured in primi- and multiparous mothers on day 4 after birth, no significant difference was obtained (ANOVA F_1,141 _= 0.53, p value = 0.47). The mean milk volume of primiparous mothers was 225 ml (SE = 14 ml) and the mean volume of multiparous mothers was 239 ml (SE = 15 ml).

**Figure 4:**
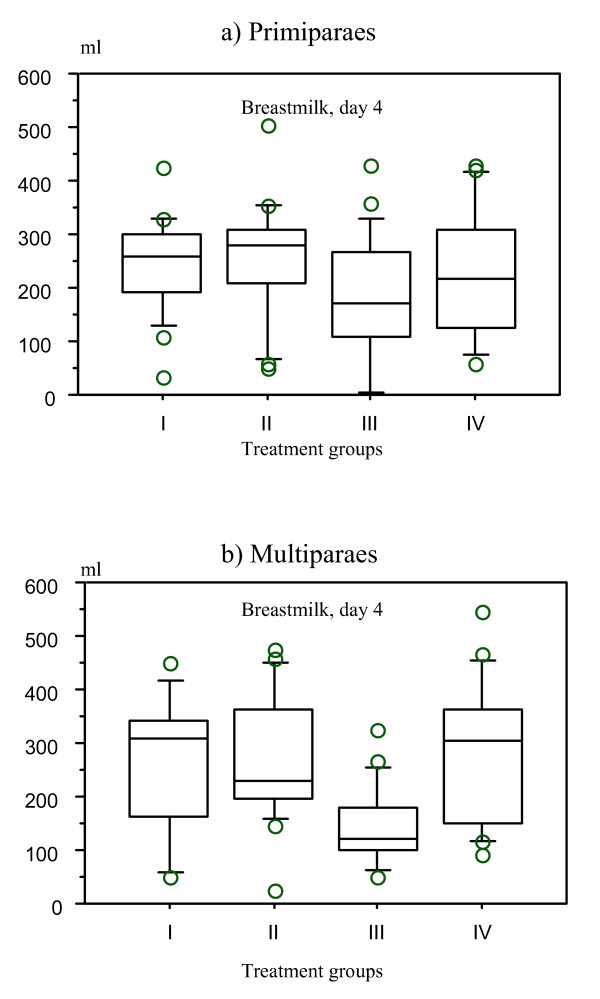
**Box plot for overall mean breastmilk volume on day 4 in four treatment groups in primi- (a) and multiparous (b) mothers**. Milk volume was lower in group III (Nursery group), but this difference was significant only in multiparous mothers (Kruskal-Wallis, H = 14.68, p value = 0.0021).

Ward routines, however, did influence the milk volume in the four treatment groups. The multiparous women in the Nursery group (Group III) had a significantly lower mean volume of milk than those in the other groups (Kruskal-Wallis, H = 14.68, p value = 0.0021). The same pattern was seen in primiparous women, but the differences did not reach significance (Kruskal-Wallis, H = 4.77, p value = 0.19) (Figure 4a, b). There was no difference in the mean amount of supplements (formula and/or glucose) given to infants of primiparas (171 ml, SE = 42 ml) and multiparas (111 ml, SE = 19 ml) in the Nursery group (ANOVA F_1,18 _= 1.97, p value = 0.18), the only group where the babies were supplemented.

#### Number of breastfeeds – days 1–3

**Figure 5:**
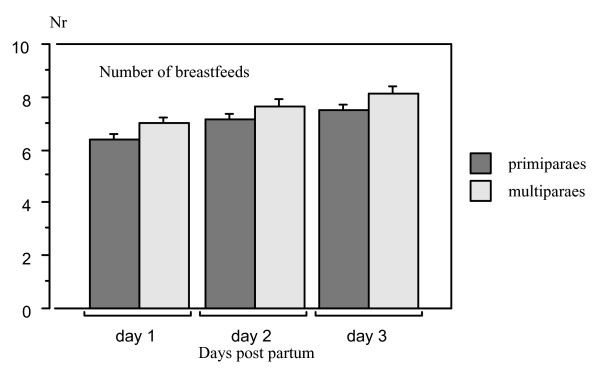
**Bar plot for overall mean number of breastfeeds during day 1 – day 3 in all mothers belonging to the three rooming-in groups (means and SE)**. The number of breastfeeds was significantly higher in multiparous mothers (repeated measures ANOVA, F_1,108 _= 6.57, p value = 0.0117 and Fisher's PLSD p values 0.0363 – 0.0001).

The number of breastfeeds in the three rooming-in groups where the infants had the opportunity to suckle on demand (Skin-to-skin, Mother's arms and Reunion groups) did not differ significantly between the groups during days 1 – 3 after birth (repeated measures ANOVA F_2,107 _= 1.42, p value = 0.25), but rose significantly as means from 6.4 to 8.6 (SE 0.28 and 0.30, respectively) during this time period. The number of breastfeeds was significantly larger in multiparous mothers (repeated measures ANOVA F_1,108 _= 6.571, p value = 0.0117 and Fisher's PLSD p values 0.0363 – 0.0001) (Figure 5).

#### Physiological breast engorgement – days 1–3

**Figure 6:**
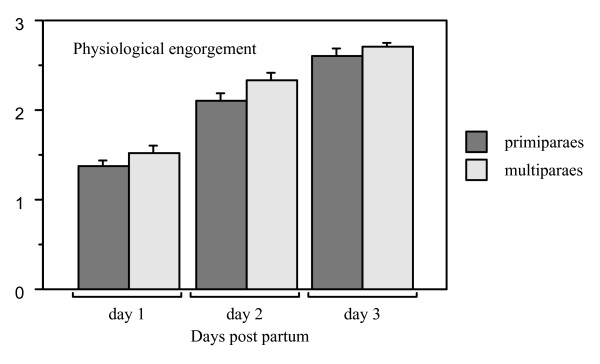
**Bar plot for estimation of physiological breast engorgement (described by the mothers as "breast fullness/tension") during day 1 – day 3 in all mothers (means and SE)**. The perception of breast engorgement was significantly higher in multiparous mothers (repeated measures ANOVA F_1,145 _= 4.10, p value = 0.0446).

The intensity of mother's perception of breast engorgement (classified as "none", "slight" or "marked") increased from one to three days after birth and was significantly more pronounced in multiparous women (repeated measures ANOVA F_1,145 _= 4.10, p value = 0.0446) (Figure 6). However, ward routines did not influence the mother's perception of breast engorgement during the time studied and there were no significant differences among the four groups (repeated measures ANOVA F_3,143 _= 0.66, p value = 0.58).

#### Feeling "low/blue" – days 1–3

**Figure 7:**
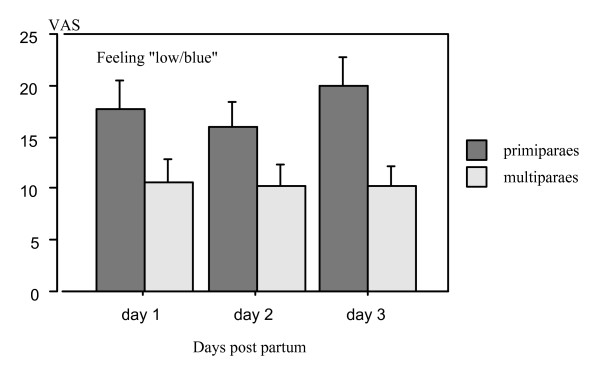
**Bar plot for perception of feeling "low/blue" (Visual Analogue Scale) during day 1 – day 3 in all mothers (means and SE)**. The feeling "low/blue" was significantly higher graded by primiparous mothers than by multiparous mothers (repeated measures ANOVA F_1,146 _= 7.57, p value = 0.0067).

The mean level of the mother's estimated intensity of feeling "low/blue" was stable during days 1–3 (repeated measures ANOVA F_147 _= 0.70, p value = 0.50). However primiparous mothers had a significantly higher mean level than multiparous over the days (repeated measures ANOVA F_1,146 _= 7.571, p value = 0.0067) (Figure 7).

### Effect of early suckling

The babies in the Skin-to-skin and the Mother's arms groups (Groups I and II) had the opportunity to suckle during the first two hours after birth. Twenty-six out of 37 infants (70%) in the Skin-to-skin group and 30 out of 40 infants (75%) in the Mother's arms group suckled the breast.

**Figure 8:**
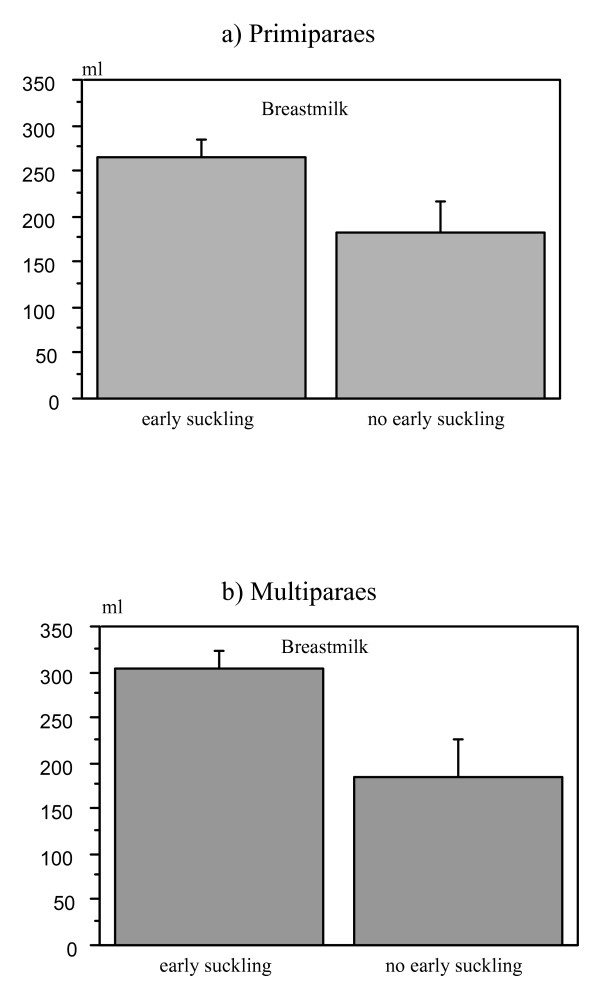
**Bar plot for overall mean breast-milk volume on day 4 in two treatment groups (Skin-to-skin gr. and Mother's arms gr.) in primi- (a) and multiparous (b) mothers, who's babies were exposed or not to early suckling (suckling within first two hours after birth) (means and SE)**. Milk volume was significantly higher if babies had early suckling, both in primiparous (ANOVA F_1,34 _= 4.63, p value = 0.0386) and multiparous mothers (ANOVA F_1,33 _= 9.0, p value = 0.0051).

Mothers of infants who suckled within the first two hours (n = 56) had significantly more milk on day 4 than mothers of infants who did not suckle (n = 21). The mean milk volume was 284 ml (SE = 14 ml) vs. 184 ml (SE = 27 ml), respectively (p value = 0.0006). There was no significant difference between primi- and multiparous women in this respect (data not shown) (Figure 8a, b).

#### Early suckling, birth weight and gestational age

Infants who suckled during the first two hours after delivery weighed significantly more at birth than those who did not suckle, 3547.9 g (SE = 62.3) versus 3290.5 g (SE = 88.7) (ANOVA F _1,75 _= 4.98, p value = 0.0286), and additionally had a higher mean gestational age of 39.6 weeks (SE = 0.1) versus 39.1 weeks (SE = 0.2) than those who did not suckle (ANOVA F _1,75 _= 4. 86, p value = 0.0305).

#### Early suckling and other variables, measured on day 3

There was no relation between early suckling and the number of breastfeeds at day 3 (ANOVA F_1,74 _= 0.06, p value = 0.81), the mothers' perception of breast engorgement at day 3 (ANOVA F_1,74 _= 0.22, p value = 0.64) or feelings of being "low/blue" at day 3 (ANOVA F_1,74 _= 0.05, p value = 0.82) in either the primiparous or multiparous mothers.

### The relative role of variables affecting milk volume on day 4

Early suckling, number of breastfeeds per day, physiological breast engorgement and maternal mood are variables known to be involved in initiation of milk production. To explore which of these variables were affecting milk production in combination with the design variables used in the present study, four multiple regression analyses were performed. The dependent variable was *amount of milk on day 4*. The explanatory variables were *early suckling *(suckling within the first two hours or not), *marked engorgement on day 3 *(marked engorgement or not during day 3), *feeling "low/blue" *on day 3 (less or more than 40 on a VAS), *number of breastfeeds *during day 3, and the three design variables *skin-to-skin *or not (baby skin-to-skin to mother or dressed in mothers arms at the delivery ward), *rooming-in *at the maternity ward or not (whether the baby stayed with the mother or in the nursery) and *swaddling *or not (whether the baby was swaddled or dressed in baby clothes). The regression coefficients estimated are given in Table 4 and Table 5.

**Table 4:** Multiple regression analyses on the dependent variable of milk amount on day 4 after delivery (72–96 hours post partum) in the mothers and babies staying together during the first 2 hours after birth: Skin-to-skin group (Group I) and Mother's arms group (Group II)

	Primiparas (n = 35; R^2 ^= 0.48)			Multiparas (n = 35; R^2 ^= 0.30)		
	
	b coefficient	std. error	p - value	b coefficient	std. error	p - value
Swaddling/clothes*	20.756	28.902	0.48	8.224	40.297	0.84
Skin-to-skin or not*	-36.466	31.898	0.26	19.181	41.582	0.65
Early suckling or not*	98.007	35.327	0.0097	123.641	43.332	0.0080
Nr of breastfeeds on day 3	20.591	7.636	0.0117	14.356	9.551	0.14
Marked engorgement on day 3*	70.749	29.377	0.0229	35.659	44.017	0.42
Feeling low/blue on day 3*	-79.356	38.339	0.0478	-71.234	85.626	0.41

*dichotomized variable

**Table 5:** Multiple regression analyses on the dependent variable of milk amount on day 4 after delivery (72–96 hours post partum) in the mothers and babies separated during the first 2 hours after birth: Nursery group (Group III) and Reunion group (Group IV)

	Primiparas (n = 35; R^2 ^= 0.42)			Multiparas (n = 35; R^2 ^= 0.43)		
	
	b coefficient	std. error	p - value	b coefficient	std. error	p - value
Swaddling/clothes*	-26.881	33.980	0.44	5.590	39.809	0.89
Rooming-in/nursery*	34.456	33.811	0.32	148.848	36.906	0.0003
Marked engorgement on day 3*	156.800	40.930	0.0006	-13.381	44.076	0.76
Feeling low/blue on day 3*	-159.050	53.980	0.0062	-102.110	63.616	0.12

dichotomized variable

For mothers and babies staying together at the delivery ward and who later had rooming-in in the maternity ward (Skin-to-skin and Mother's arms groups), the explanatory variables "skin-to-skin", "swaddling", "early suckling", "marked engorgement on day 3", "feeling 'low/blue' on day 3" and "number of breastfeeds on day 3" were used in the regression analyses. The regression analysis performed in primiparas showed that the most important variable for milk volume on day 4 was "early suckling". According to the b-coefficient, successful early suckling was associated with a mean increase of 98 ml of milk (keeping other variables constant). In addition, marked breast engorgement on day 3 was associated, on average, with 71 ml more milk and each extra breastfeed on day 3 resulted in a mean increase in milk amount of 21 ml. In contrast "feeling 'low/blue' on day 3" resulted in a mean decrease of 79 ml of milk.

When the same regression was performed in the multiparous mothers of the above mentioned groups, the only significant explanatory variable was "early suckling", which was associated with an increase of 124 ml milk on day 4.

In the two groups of mothers who did not stay together with their babies at the delivery ward, but who were exposed to a two hour separation period, one group was allowed rooming-in at the maternity ward (Reunion group, Group IV) while infants in the other group remained in a nursery (Nursery group, Group III) during the remaining time spent at the maternity home. For these two groups, the explanatory variables "rooming-in", "swaddling", "marked engorgement on day 3" and "feeling 'low/blue' on day 3" were used in the regression analyses, performed in primi- and multiparous women, respectively. Again different results were obtained in primi- and multiparous mothers. The variable "marked engorgement" was associated with a significant mean increase of 157 ml of milk on day 4 while "feeling low/blue" gave a significant mean decrease of 159 ml in the primiparous mothers. In the multiparous mothers, however, the only significant explanatory variable was "rooming-in" which was associated with a mean increase of 149 ml milk.

### Long-term effects of parity and ward routines on duration of "nearly exclusive" breastfeeding

The only variable found to predict the duration of "nearly exclusive" breastfeeding was "amount of milk ingested on day 4". There was a significant correlation between the variables "amount of milk ingested on day 4" and the "total time of 'nearly exclusive' breastfeeding" (simple regression, b = 0.275, F_1,132 _= 21.6, R^2 ^= 0.143, p value < 0.0001). Thus, the more milk produced on day 4, the longer the duration of "nearly exclusive" breastfeeding. The value of the estimated beta-coefficient (0.275 days per/ml) indicates that it can be predicted that per 100 ml of milk produced on day 4, the mothers will, on average, breastfeed almost a month longer.

**Figure 9:**
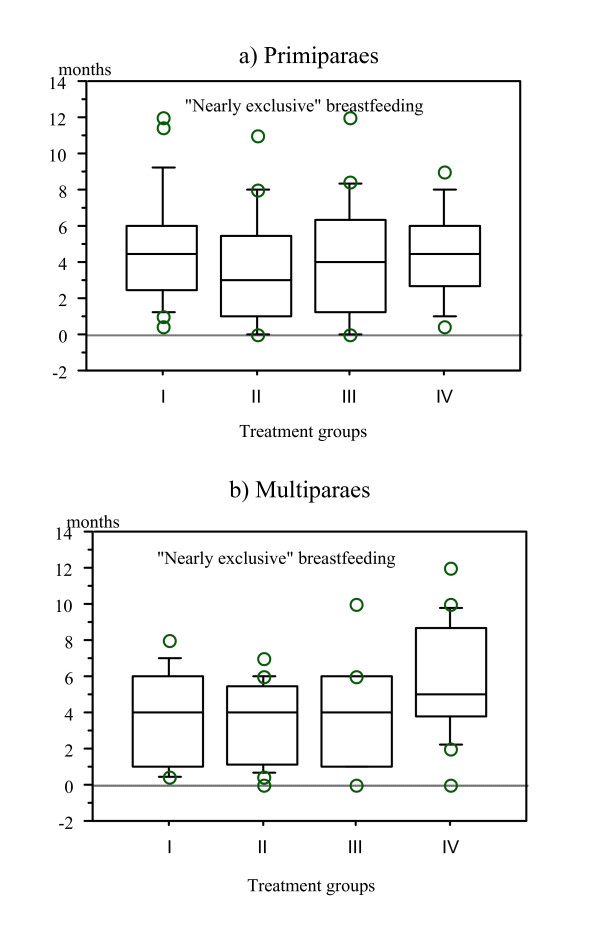
**Box plot for the duration of "nearly exclusive" breastfeeding in four treatment groups in primi- (a) and multiparous (b) mothers**. There was no significant differences in the duration of "nearly exclusive" breastfeeding between groups both in primi- and multiparous mothers.

The median duration of "nearly exclusive" breastfeeding was 4.0 months overall and there was no significant difference between the four treatment groups either in the primiparous (Kruskal-Wallis, H = 1.66, p value = 0.65) or the multiparous mothers (Kruskal-Wallis, H = 5.90, p value = 0.12) (Figure 9a, b). No difference could be demonstrated in duration of "nearly exclusive" breastfeeding between babies who did or did not suckle during the first two hours after birth (p value = 0.25). Nor could any relationship between parity, intensity of breast engorgement, number of breastfeeds or feelings of being "low/blue" be demonstrated.

## Discussion

The main findings in the present study were that ward routines/practices at both the labour and the maternity ward influenced milk ingestion/production four days after birth. In addition, maternal experience of breast engorgement and feeling "low/blue" were related to milk ingestion/production. The influence of these variables differed between primi- and multiparous women.

In the current study, milk production/ingestion four days after birth or the duration of "nearly exclusive breastfeeding" did not differ between the groups allowed skin-to-skin contact, being in mother's arms dressed or swaddled, or exposed to a short-term separation after birth. In contrast, the procedure of early suckling was associated with an increased milk ingestion/production four days after birth.

In some, but not all, previous studies [7,18], positive effects of early skin-to-skin contact on the duration of breastfeeding have been demonstrated [19-22]. Early suckling has also been shown to promote breastfeeding [19,23-25]. The different results obtained between studies may be related to the fact that the design of the previous studies did not always allow a clear distinction between the effects of skin-to-skin contact and early suckling on the outcome of breastfeeding.

It should be mentioned, however, that the infants in our study that suckled early weighed significantly more and had a significantly higher mean gestational age than those that did not suckle, differences which may be related to the outcome.

Surprisingly the amount of milk ingested/produced four days after birth did not differ between the groups of mothers and infants that were allowed to stay together after birth (Skin-to-skin and Mother's arms groups) or that were subjected to a short-term separation (Reunion group), in spite of the positive effect of early suckling in the former groups.

Maternity ward routines had a strong impact on the outcome of milk ingestion/production in the present study. Infants staying in the nursery at the maternity ward ingested less milk than infants who were rooming-in and thus allowed breastfeeding on demand. This difference in milk ingestion reached significance in infants of multiparous women. This lower milk intake was not related to more supplementation.

The difference in the amount of milk ingested/produced on day four may be related to the fact that the number of breastfeeds was set to seven times per day in the group of mothers who had their infants in the nursery (Group III), to the use of infant formula in the nursery, and most likely to the more limited contact between mother and infant subjected to this routine due to the separation of mother and infant in between feeds.

Swaddling did not have any significant effect on milk production as had been hypothesised, since in a previous study we found that swaddling could delay recovery of weight loss in babies while combined with potential stressors, such as separation after birth and supplementation by formula or glucose [10].

The relative importance of the different parameters shown to be of importance for milk ingestion/production four days after birth was further investigated by means of regression analysis. The design of the study allowed four different analyses to be performed. Thus, groups of mothers and infants that were staying together at the labour ward and those that were separated at this time were analysed separately and there was a further split of the data based on parity (Table 4 and Table 5).

In the groups of mothers and infants that stayed together in the labour ward, early suckling, the intensity of breast engorgement and the number of breastfeeds on day 3 after delivery were positively associated, and the intensity of feeling "low/blue" was negatively associated, with the amount of milk ingested/produced four days after birth. These relationships were, however, only seen in the primiparous mothers. In multiparous mothers, early suckling was found to be the only factor of importance.

A similar pattern was obtained in the mothers and infants that were exposed to a short-term separation after birth. Thus, even in the separated groups, the intensity of breast engorgement was positively associated and the intensity of feeling "low/blue" was negatively associated with the amount of milk ingested/produced four days after birth in the primiparous women, whereas the routine of rooming-in was of singular importance for milk ingestion/production in multiparous women.

One conclusion that could be drawn from the regression analyses is that milk production might be differently influenced in primi- and multiparous women. Obviously, the intensity of breast engorgement was associated with milk ingestion/production in primiparous mothers, irrespective of labour ward routines, whereas multiparous mothers seemed to rely more on "sensory stimulation", such as the procedure of early suckling or the routine of rooming-in.

Breast engorgement has been suggested to be related to "hormonal" stimulation of milk production. Oestrogen and progesterone levels are high during pregnancy, but since these hormones are, to a great extent, produced in the placenta, the levels of these hormones decrease rapidly after birth. Decreased levels of these steroid hormones have been associated with initiation of milk production [26,27]. Possibly the experience of physiological engorgement is related to this phenomenon.

Oxytocin is of main importance for milk ejection and related behaviours [28]. Animal studies indicate that there are differences in primi- and multiparas regarding sensitivity to sensory cues that trigger milk ejection and maternal behaviour. Animal studies show that in ewes, the first pregnancy induces permanent changes with regard to the release of oxytocin and oxytocin receptors, e.g. the expression of oxytocin receptor mRNA is increased in the paraventricular nucleus (PVN) of the brain and the sensitivity of oxytocin autoreceptors is increased [29]. These changes resulting from maternal experience may facilitate the expression of oxytocin-mediated functions, such as milk ejection, in connection with later pregnancies. An analogous finding is that the prolactin surge to suckling is blunted in rats having previous experience of lactation [30]. Studies in humans actually indicate a similar increased sensitivity of prolactin receptors in multiparous when compared to primiparous women [31].

These data may indicate that the "machinery" for milk production might be primed by previous lactations also in women. As a consequence, it may be easier to trigger milk production by sensory stimuli. Thus, early suckling and/or rooming-in may suffice to initiate milk production, when the mammary tissue is already primed.

The results of the multiple regressions must, however, be interpreted with some caution. Since they were based on information obtained on days 3 and 4 of lactation, it cannot be excluded that other relationships might have been established if data from other time points had been explored. Other unknown factors may also play an important role. Still the similarity of the results obtained for primiparous women in the regressions performed on the groups of mothers and infants that stayed together after birth and those that were separated after birth speak strongly in favour of the reliability of the analyses.

In the present study we were able to cover the impact of some factors affecting milk ingestion/production four days after birth. This may not complete all possible influences such as cultural differences, for example. Yet the above data suggest that milk production, a physiological function of vital importance for the offspring and survival of the species, is under multifactorial control. When infants are allowed close contact with their mother immediately after birth, early suckling may be a strong promoter of milk production. If, on the other hand, mother and infant are separated after birth, e.g. because of complicated delivery, alternative physiologic pathways may be activated to ascertain an adequate production of milk. Intense sensory stimulation as offered by the routine of rooming-in or feeding on demand may compensate for effects cause by short-term early separations.

It is important to mention that early skin-to-skin contact should be promoted even if it does not seem to play a direct role for milk production and duration of breastfeeding. Skin-to-skin contact in this study started between 20 – 25 minutes after birth as the infants were subjected earlier to compulsory hospital practices. This may have interfered with the baby's developing breastfeeding behaviour [1] and may have disguised some effects of immediate skin-to-skin contact.

Even if there are no measurable effects of skin-to-skin contact, it may still create a positive experience for the infant and the mother and facilitate the procedure of breastfeeding [1,32]. Additionally, stress levels have been shown to be reduced both in mothers and infants [3,4], and, as has been demonstrated by Klaus and Kennell [33] amongst others, early skin-to-skin contact occurring immediately after birth may promote attachment between mother and child and facilitate maternal-infant interaction in a long-term perspective.

## Conclusion

Our study shows that ward routines influence milk production. The data also suggest that milk production in primi- and multiparous women may be differently influenced or even regulated by many factors and further research to identify these factors is needed.

## Competing interests

The author(s) declare that they have no competing interests.

## Authors' contributions

KB collected, coded and analysed data; performed statistical analyses; drafted the manuscript. A-MW conceived the study, and developed its design; coordinated the study; collected and analysed data; drafted the manuscript; performed statistical analyses. A-SM participated in its design; collected data; revised the manuscript; performed statistical analyses. A-BR-A conceived the study and participated in its design; collected data; revised the manuscript. BW-N planned the study; collected data; revised the manuscript. IV conceived, planned and organized the study. KU-M analysed data; developed theoretical perspective; drafted the manuscript.
